# Assessing the determinants of uptake and hesitancy in accessing COVID 19 vaccines in Nigeria: a scoping review

**DOI:** 10.3389/frhs.2025.1609418

**Published:** 2025-09-04

**Authors:** Chikezie Ifeanyi, Emmanuel Okechukwu, Olushola Tosin, Ichoku Hyacinth, John Ele-Ojo Ataguba, Grace Njeri Muriithi, Daniel Malik Achala, Elizabeth Naa Adukwei Adote, Chinyere Ojiugo Mbachu, Senait Alemayehu Beshah, Chijioke Osinachi Nwosu, John Thato Tlhakanelo, James Akazili, Nyasha Masuka

**Affiliations:** ^1^ Health Systems and Development Research Group, Veritas University Abuja Nigeria, Abuja, ACT, Nigeria; ^2^ African Health Economics and Policy Association (AfHEA), Accra, Ghana; ^3^ Department of Community Health Sciences, Max Rady College of Medicine, Rady Faculty of Health Sciences, University of Manitoba, Winnipeg, Manitoba, Canada; ^4^ Partnership for Economic Policy (PEP), Nairobi, Kenya; ^5^ School of Health Systems and Public Health, University of Pretoria, Pretoria, South Africa; ^6^ Department of Community Medicine, University of Nigeria, Enugu, Nigeria; ^7^ Ethiopian Public Health Institute, Addis Ababa, Ethiopia; ^8^ Department of Economics and Finance, University of the Free State, Bloemfontein, South Africa; ^9^ Department of Family Medicine and Public Health, Faculty of Medicine, University of Botswana, Gaborone, Botwana; ^10^ School of Public Health, C.K Tedam University of Technology and Applied Sciences, Accra, Ghana; ^11^ Zimbabwe College of Public Health Physicians, Harare, Zimbabwe

**Keywords:** COVID-19, acceptance, hesitancy, uptake, Nigeria, scoping review

## Abstract

The coronavirus disease (COVID-19) is one of the largest public health threats in recent times, with significant health, economic, and social consequences globally. The WHO reported that over 651 million cases and 6.6 million deaths were attributed to COVID-19 globally. The Nigeria Centre for Disease Control (NCDC) in 2022 revealed that 266,057 cases with 3,155 deaths were reported. All the thirty-six states and the Federal Capital Territory (FCT) of Nigeria were affected, but Lagos and the FCT reported the highest number of cases. However, it is possible that these numbers do not accurately reflect the severity of COVID-19 disease in Nigeria because the country had only tested 5,160,280 people as at 2022, despite a population of around 200 million. Nigeria did not meet its 2021 vaccination target, prompting the need to identify the contextual factors affecting vaccine access and uptake as well as vaccine hesitancy in Nigeria and document the approaches that can be deployed to reduce opposition to vaccination as well as improve advocacy for vaccine equity. This scoping review, conducted using Arksey and O'Malley's framework, aimed to explore the factors influencing COVID-19 vaccine hesitancy and uptake in Nigeria. A comprehensive literature search was conducted across electronic databases, including Google Scholar and PubMed, with studies from Nigeria published in English. The review included 25 studies on vaccine hesitancy, uptake, and willingness to accept COVID-19 vaccination, identifying barriers at the national, community, and individual levels. The results indicated that 90% of the studies showed low vaccine acceptance and uptake, with barriers related to vaccine availability, misinformation, cultural and religious influences, socioeconomic factors, and lack of trust in the health system. Socio-demographic factors such as gender, age, education, and income were identified as key influences. The findings highlight the need for targeted, evidence-based strategies to address vaccine hesitancy, improve vaccine distribution, and engage diverse population groups to enhance vaccination uptake across Nigeria.

## 1 Introduction

Coronavirus disease (COVID-19) is one of the largest public health concerns recently. It is connected with massive health, economic and social consequences, globally (1). The World Health Organization (WHO) officially declared the pandemic a public health emergency of international concern by the end of January 2020 (2). The pandemic had huge socio-economic consequences worldwide. For instance, the COVID-19 lockdown periods were associated with a 34.1% economic loss amounting to USD 16 billion in Nigeria's Gross Domestic Product (GDP) with the services and agricultural sectors being the worst hit (3). Furthermore, about 60% of Nigerians were food insecure and this worsened due to the adverse impact of COVID-19 (4).Therefore, COVID-19 has multidimensional impacts on health, food security, and the economy (5).

The WHO reported that over 651 million cases and 6.6 million deaths were attributed to COVID-19 globally (6). The Nigeria Centre for Disease Control (NCDC) in 2022-week 46 update revealed that 266,057 cases with 3,155 deaths were reported (7). All the thirty-six states and the Federal Capital Territory (FCT) of Nigeria were affected, but Lagos and the FCT reported the highest number of cases (7). However, it is possible that these numbers do not accurately reflect the severity of COVID-19 disease in Nigeria because the country had only tested 5,160,280 people as at May 2022, despite a population of around 200 million (8).

However, the trend of spread of COVID-19 is being reversed with the introduction of COVID-19 vaccines across the globe. A number of COVID-19 vaccines have been developed and introduced to minimize the risk of COVID-19 related deaths (7). The WHO approved 11 vaccines for emergency use listing^1^, while Nigeria has approved seven, namely: Vaxveria (Oxford/AstraZeneca), Covishield (Serum Institute of India), Comirnaty (Pfizer/BioNTech), Jcovden (Johnson and Johnson) and Spikevax (Moderna), Sputnik V (Gamaleya) and Covilo (Sinopharm) (9).

Nigeria did not attain the December 2021 vaccination target of 40 per cent. According to the National Primary Health Care Development Agency (NPHCDA), the number of fully vaccinated individuals was 9.8 per cent (10,925,624) as of, 2022. Health experts were concerned that the country did not meet the 70 per cent target by June 2022 (8). Similarly, global data indicated that only 13% of the Nigerian population had been fully vaccinated. Furthermore, studies conducted in Northern Nigeria reported that acceptance of the COVID-19 vaccine was less than optimal (10). Also, a cross-sectional survey conducted at a university in the eastern part of Nigeria reported a COVID-19 vaccine hesitancy rate of 65.04% (11).

Several studies have looked at the factors that contribute to COVID-19 vaccine hesitancy: low educational attainment, ethnic disparities, rurality, and resistance to other vaccinations (e.g., influenza) were reported common variables (12, 13). At first, the inadequate supply of COVID-19 vaccines in Nigeria was the primary reason for the country's low vaccination rate, since the vaccines were imported. However, with an increase in supply, the supply-and-demand dynamic took effect, and vaccine acceptance became a crucial factor in determining coverage. Low COVID-19 vaccine uptake has significant negative consequences, which cannot be overemphasized. For instance, achieving herd immunity to disrupt the spread of the infection is nearly impossible without a high vaccination rate. For herd immunity to be achieved based on current estimates, more than 60% of the population would need to have either a natural COVID-19 infection or vaccination (14).

In order to identify the key contextual issues and proffer recommendations to promote equitable vaccine access, improve vaccine uptake, and reduce vaccine hesitancy there is need to undertake the study in Nigeria. This study synthesizes existing literature on COVID-19 vaccine access and hesitancy in Nigeria to explore enablers and barriers to equitable and timely vaccine access to aid decision and inform policies.

## 2 Materials and methods

### 2.1 Study design

This scoping review was conducted adopting Arksey and O'Malley's framework (15). The framework consists of five phases: a. formulating the research questions; b. conducting a comprehensive literature search; c. selecting relevant studies; d. extracting and organizing data; and e. synthesizing and reporting the results. This review followed the Preferred Reporting Items for Systematic Review and Meta-Analysis extension for Scoping Reviews (PRISMA-ScR) checklist (16).

### 2.2 Search engines and strategies

Literature search was conducted on the following electronic databases: Google Scholar, African Journals Online, PubMed, and Science Direct, using a combination of keywords and appropriate lexica for COVID-19 vaccine, vaccination, access/uptake and hesitancy. The keywords were conveniently selected based on the study objectives: vaccine, prevalence, COVID-19, Nigeria, sub-Saharan Africa, uptake, access, hesitancy, refusal, acceptance, and acceptability. The literature search was supplemented with resources from institutional sources. For all databases, the search followed a systematic approach that utilized specific focus areas, search queries, and criteria (See Table 1).

**Table 1:** Literature search words/phrases/sentences.

SN	Cases	Review query
1	Focus	‘’COVD-19’’, SAR-Cov 2
2	Action	Vaccines, vaccination OR immunization
3	Action	‘’acceptance rate’’ OR hesitancy OR ‘’Hesitancy rate’
4	Factors	Enablers, Barriers, Predictors
5	Location	Nigeria Africa, Global
6	Phrase together	1 AND 2 AND 3 AND 4 AND 5
7	Timelines	December 2020 to December, 2023
8	Language	Limited to English
9	Combination of words/phrases	6 AND 7 AND 8

**Table 5:** Enablers, barriers, and facilitators of vaccination including COVID 19 uptakes in Nigeria.

Levels	Enablers	Barriers
National/States/LGAs	• Supporting NGOs and partners • Coordinating MDAs • Nationwide vaccine programme (NPSCMP) • Point of entry and holding • Policies • Health surveillance system	• Vaccine shortage • Pre-existing social inequalities • Complexity of Nigeria distribution and allocation systems
Health Systems	• Availability of public & private health facilities (tertiary, secondary and primary) • Human resources for health • Immunization data base • Health education and trainings • Pre-existing monitoring systems • Free vaccination for All	• Funding • Poor health infrastructure and equipment • Long waiting periods • Follow up challenges
Community	• Social contacts (information from families and friends) • Community gatekeepers • Religious leaders • Health promotion and outreaches	• Religious influences • Cultural inclination • Myths • Residential segregation
Individual and Family	• Parental decisions • Friends and families—communications • Previous experience—vaccination status • Risk factors • Awareness creations • Health Insurance • Education attainment • Employment • Funds • Certificates/travelling purposes • Opening the economy	• Distance to health facilities • Finances (socio-economic status)—Transportation challenges • Lack of faith in vaccines • Fear of side effects and complications • Information/education, awareness • Never being offered before • Low literacy levels • Age • Gender

Sources (17, 30, 31).

### 2.3 Study exclusion and inclusion criteria

In totality, studies conducted in English about COVID-19 vaccination, access and uptake plus hesitancy with special emphasis on Nigeria since December 2020 were considered. Our inclusion criteria included only studies reporting access, uptake, and COVID-19 vaccination hesitancy published, relevant articles published from December 2020 to December 2023. The study excluded COVID-19 studies reporting animal studies or reviews, commentaries, and others not related to vaccine access/uptake and hesitancy amongst humans.

### 2.4 Quality appraisal of selected literature

The Joanna Briggs Institute (JBI) framework was employed for assessing the study quality. We developed quality assessment criteria with categories of low, medium, and high (See Tables 2–4) study quality for assessing access, uptake, and barriers to equitable and timely uptake of COVID-19 vaccines, as well as strategies for addressing these barriers among disadvantaged groups in Nigeria.

**Table 2:** Uptake and coverage of COVID 19 vaccines.

Author	Title	Region	Methodology	Study participants	Year of Publication	Sample Size	Quality rating	Coverage/Uptake rate
Obianuju et al.	Enablers and barriers to COVID-19 vaccine uptake in an urban slum in Lagos, Nigeria: informing vaccine engagement strategies for the marginalized	Lagos - Nigeria	Population based case control	Adult urban slum dwellers	2022	45	High	<10%
Suleiman Zakari et al.		Benue Nigeria	A web-based cross cross-sectional survey	staff and students at Federal University of Health Sciences	2023	150	Medium	3%
Damian et al.	Factors Influencing the Intention and Uptake of COVID-19 Vaccines on the African Continent: A Scoping Review	Africa	Systematic Review	General population	2023	40	High	
Dunkwu et al.	COVID-19 Vaccine Uptake and Hesitancy amongst University Students in a Tertiary Institution in Edo State, Nigeria	Edo - Nigeria	Cross sectional	University Students	2023	677	High	9.2% (62/677)
Babatope et al.	COVID-19 vaccine hesitancy: a systematic review of barriers to the uptake of COVID-19 vaccine among adults in Nigeria.	Nigeria	A systematic search of indexed electronic peer-reviewed literature published from 2019 onwards	Adult population	2023	148	High	24.3% to 49.5% amongst high risk population and 26% amongst low risk population
Iwuagwu et al.	Why I have not taken the COVID-19 vaccine” a descriptive qualitative study of older adults’ perceived views of COVID-19 vaccine uptake in Nigeria.	Nigeria	Qualitative method—Cross sectional	older adults	2023	16	High	
Gilbert et al.	Covid-19 vaccine hesitancy and associated factors among adults in urban and rural communities in rivers state.	Rivers State, Nigeria	comparative cross-sectional study	Adult population	2023	422	High	13.7%
Shallangwa et al.	Assessment of COVID-19 vaccine hesitancy among people living with HIV/AIDS: a single-centered study.	Maiduguri, Borno State, Nigeria.	A hospital-based cross-sectional study design	older adults	2023	344	High	10.5%(27/256)

**Table 3:** Factors and predictors of COVID 19 hesitancy.

Author	Title	Region	Study design	Study participants	Year	Sample size	Quality rating	Key finding
Shallangwa et al.	Assessment of COVID-19 vaccine hesitancy among people living with HIV/AIDS: a single-centered study.	Maiduguri, Borno State, Nigeria.	A hospital-based cross-sectional study design	older adults	2023	344	High	• low effectiveness of the COVID-19 vaccine • low level of knowledge • safety concerns • lack of trust on stakeholders • low vaccine efficacy
Obianuju et al.	Enablers and barriers to COVID-19 vaccine uptake in an urban slum in Lagos, Nigeria: informing vaccine engagement strategies for the marginalized	Lagos - Nigeria	Population based case control	Adult urban slum dwellers	2022	45	High	• Perceived low effectiveness of the COVID-19 vaccine among • low level of knowledge. • Poor access, safety concerns, • lack of trust, low vaccine efficacy • low susceptibility
Babatunde et al.	COVID-19 vaccine hesitancy in six geopolitical zones in Nigeria: a cross-sectional survey	Nigeria – 6 Geopolitical zones	Cross sectional	Healthcare workers	2022	1615	High	• Traditional homogeneity and previously experienced hesitancy • Location
Dunkwu et al.	COVID-19 Vaccine Uptake and Hesitancy amongst University Students in a Tertiary Institution in Edo State, Nigeria	Edo – Nigeria	Cross sectional	University Students	2023	677	High	• Age, Gender, Educational Level
Gilbert et al.	Covid-19 vaccine hesitancy and associated factors among adults in urban and rural communities in rivers state.	Rivers State, Nigeria	comparative cross-sectional study	Adult population	2023	422	High	• Location • Gender
Adeniyi, D.S.	Drivers of Covid-19 Vaccine Hesitancy in Southern Nigeria.	Ibadan Nigeria	Cross sectional survey		2022	1500	High	• lacks of adequate information • skeptism about the safety of the Covid-19 vaccines
Chutiyami, M et al.	Subjective reasons for COVID-19 vaccine hesitancy and socio-demographic predictors of vaccination in Nigeria	Nigeria	An online social media survey, analyzed	Adult population	2022	576	High	• Educational level • Gender • Misinformation • Location
Zakari. et a.l	Acceptance and hesitancy of COVID-19 vaccine among university community members of Otukpo, Nigeria: a cross-sectional study.	Benue Nigeria	A web-based cross-sectional survey	Adult population	2023	150	High	• skepticism about the vaccination due to fast production and rollout • Fear of vaccine side effects
Ogunbosi, B. O., et al	COVID-19 vaccine hesitancy in six geopolitical zones in Nigeria: a cross sectional survey.	Nigeria	A cross sectional survey	Adult population	2022	1615	High	• Location
Ogunbosi et al.	COVID-19 vaccine hesitancy in six geopolitical zones in Nigeria: a cross sectional survey.	Nigeria	A cross sectional survey	Adult population	2022	1,615	High	•, Location
Aseneh et al.	Factors associated with COVID-19 vaccine hesitancy among healthcare workers in Cameroon and Nigeria: a web-based cross-sectional study.	Cameroon and Nigeria	A cross sectional survey	Adult population	2023	598	High	• Little or no trust on efficacy • Side effects • Uncertainty
Adigwe et al.	COVID-19 vaccine hesitancy and willingness to pay: Emergent factors from a cross-sectional study in Nigeria.	Nigeria	A cross sectional survey	Adult population	2021	1,767	High	• Side effect • Financial implications
Ojewale, L.Y. & Mukumbang, F.C.	COVID-19 vaccine hesitancy among Nigerians living with noncommunicable diseases: a qualitative study.	Ibadan, Nigeria	Qualitative method—Cross sectional	Adult population	2023	25	High	• Concerns over the COVID-19 vaccine worsening the underlying chronic condition; • Fear of harmful physiological consequence • Concerns over insufficient testing of vaccine for safety • Perceived vaccine infectiveness
Eguavoen et al.	Reducing COVID-19 vaccine hesitancy and improving vaccine uptake in Nigeria	Nigeria	Qualitative method—Cross sectional	Adult population	2023	523	High	• Concerns around vaccine efficacy and safety, • Disbelief in the existence and severity of the disease, • Distrust of the government.
Zwawua, O. & Kor, E.	Factors Associated with COVID-19 Vaccine Hesitancy among a Rural Sample in Benue State.	Benue State, Nigeria	Qualitative method—Cross sectional	Adult population	2022	16	High	• COVID-19 vaccines were regarded as ‘mark of the devil’ and as weapon of destruction of Africans by the western countries. • severity of COVID-19 and less vulnerability to the disease.

**Table 4:** Barriers and enablers of uptake and access to COVID 19 vaccination.

Author	Title	Region	Study design	Study participants	Sample size	Quality rating	Enablers	Barriers
Obianuju et al. (17)	Enablers and barriers to COVID-19 vaccine uptake in an urban slum in Lagos, Nigeria: informing vaccine engagement strategies for the marginalized	Lagos - Nigeria	Population based case control	Adult urban slum dwellers	45	High	• Approval from family, friends • Health workers	• Poor access, • safety concerns, • lack of trust, • low vaccine efficacy and • low susceptibility
Damian et al. (36)	Factors Influencing the Intention and Uptake of COVID-19 Vaccines on the African Continent: A Scoping Review	Africa	Systematic Review	General population	40	High	• desire to protect others, family, community members, and vulnerable people • Knowledge and awareness • Access to media	• Fears over potential side effects • Concerns regarding the vaccine's ineffectiveness in protecting against COVID-19 • The vaccine was designed to sterilize the African population • Lack of information • mistrust in science or the vaccine • vaccine inaccessibility demanding work schedules, vaccine shortages, long • queues, and hard-to-access vaccination sites, a lack of trust in stakeholders (e.g., • vaccine manufacturers and the government)
Dunkwu-Okafor et al. (18)	COVID-19 Vaccine Uptake and Hesitancy amongst University Students in a Tertiary Institution in Edo State, Nigeria	Edo - Nigeria	Cross sectional	University Students	677	High	• Immunity against infection • International travel purposes	
Josiah and Kantaris (19)	Perception of Covid 19 and acceptance of vaccine in Delta state Nigeria.	Delta State, Nigeria	Cross sectional survey	Adult population	401	High		• Possible side effects, safety, • Efficacy concerns.
Edafe and Okoro (20)	Factors associated with COVID 19 vaccine hesitancy among residents of a semi-urban setting in Bayelsa State, Nigeria.	Bayelsa State, Nigeria	descriptive cross-sectional survey	Adult population	1,100	High		• Possible side effects, safety, • Efficacy concerns.
Ojewale and Mukumban (21)	COVID-19 vaccine hesitancy among Nigerians living with noncommunicable diseases: a qualitative study.	Ibadan, Nigeria	Qualitative method—Cross sectional	Adult population	25	High		• Misconceptions of vaccines as a treatment for those with COVID-19 • Mistrust of manufacturers (‘the whites’); • Mistrust of government and • COVID-19 misinformation.
Zwawua and Kor (22)	Factors Associated with COVID-19 Vaccine Hesitancy among a Rural Sample in Benue State.	Benua State, Nigeria	Qualitative method—Cross sectional	Adult population	16	High		• COVID-19 vaccines were regarded as ‘mark of the devil’ and as weapon of destruction of Africans by the western countries.
Romate et al. (23)	What contributes to COVID-19 vaccine hesitancy? A systematic review of the psychological factors associated with COVID-19 vaccine hesitancy.	Worldwide	Systematic Review	N.A	79	High		• Vaccine safety and side effects • Vaccine confidence/trust, trust in government and healthcare professionals, • Skepticism around vaccine production, • Conspiracy beliefs, emotions, and information • Knowledge about the vaccine

### 2.5 Data extraction and processing

The inclusion and exclusion criteria guided the selection of full-text studies to determine which studies were most appropriate to include in this review. The review focused on quantitative, qualitative, and mixed-methods studies published in English language in peer-reviewed journals on access, uptake, barriers, factors, facilitators, and hesitancy toward COVID-19 vaccines in Nigeria. A standardized data extraction sheet in Microsoft Excel was used to collate and chart the data into themes, and to summarize the studies and reports See Tables 2–4. The following headings were used to extract detailed information for the included studies: authors and year of publication; study setting, i.e., country and data collection period; and methodology. The methodology section consisted of study characteristics, i.e., study design, target population, and sample size. Four reviewers (IEH, EIO, CIN and OSO) conducted data screening and extraction, and disagreements were resolved through discussions with a fourth reviewer.

## 3 Results

### 3.1 Selection of studies

**Figure 1:**
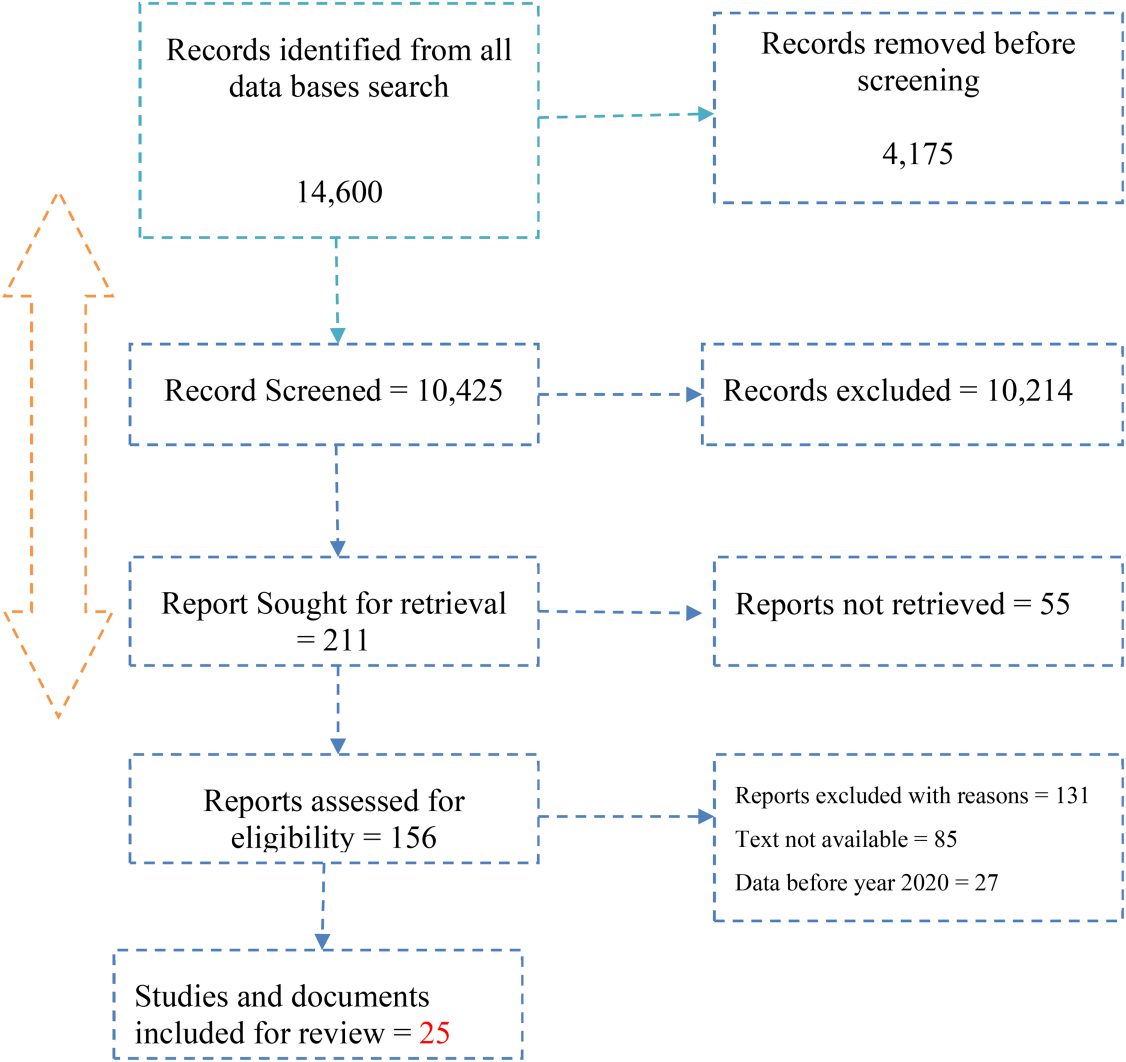
The preferred reporting items for systematic review and meta-analysis (PRISMA) flow diagram of the study selection process.

A total of 14,600 search results were identified from the different databases. After screening, removing duplicates, and other ineligible records, 10,425 studies were screened and 211 studies identified for retrieval. A total of 131 studies were identified as having data before study inclusion timeline, studies of vaccine of no interest and not available. At the end, 25 studies were included in the review and this incorporates 15 studies on COVID-19 vaccination hesitancy and 10 studies on uptake and willingness to accept COVID-19 vaccination. The scheme for selecting the studies is presented in the PRISMA flow diagram (See Figure 1).

### 3.2 COVID 19 uptake and associated factors

Ten (10) articles were included in the final analysis for vaccine acceptance, access and uptake based on the inclusion and exclusion criteria that were developed for the reviewing studies on COVID-19 acceptance, coverage and associated factors in Nigeria. There was heterogeneity in the study in terms of the study region, study design, and study participants. The studies were conducted across different regions and cities in Nigeria except one study across countries in Africa (24). For study design and participants, 82% of the studies were on general population and are cross-sectional studies conducted on adult population while 18% were institution-based studies with university staff and students as participants (18, 21, 24, 25, 17). In terms of regional differentiation, 40% of the studies were conducted in the south-south, 30% from the south western part and 30% from the northern part of the country, respectively (24).

In general, about 90% of the studies reviewed showed low acceptance and uptake of COVID-19 vaccines in Nigeria. The studies showed an average range of 9% to 24% rate of uptake with the highest rates being from hospital-based surveys with staff and patients while the lowest uptake rates were reported among respondents in rural locations of the country (19, 21, 24, 25, 17, 26).

For instance, a study conducted in Delta State showed 48.6% of the respondents were willing to take Covid-19 Vaccines (19). Also, a study conducted in Nigeria revealed that only 10.5% were willing to be vaccinated, while the majority: 57.8% (148/256) were not willing to be vaccinated and 31.7% (81/256) of the respondents were uncertain, thus resulting in a hesitancy rate of 89.45% (26). The NPHCDA in 2022 reported that about 8% of Nigeria's total population were vaccinated with the first dose while only 45% of the latter received the second vaccine dose (27). Acceptance rates ranges of 24.3% to 49.5% were reported across the four studies conducted among high-risk populations in Nigeria, while the acceptance rates among low-risk populations ranged from 26.0% to 86.2%. On the other hand, an online study conducted in Nigeria revealed that 80% of the respondents indicated willingness to get the vaccine (24). Challenges to COVID-19 Vaccine Distribution and Delivery to the Last Mile.

### 3.3 Pattern of access and uptake data

A number of empirical studies have been conducted to glean a comprehensive understanding of the patterns of access and vaccine uptake in Nigeria (18). These studies aimed to identify and address specific challenges hindering vaccination efforts. A study conducted in 2022 reported that only 32.8% of respondents had received the COVID-19 vaccine. Residents of urban areas recorded a higher level of vaccine uptake (34.4%), compared to those in rural Nigeria where the uptake was 30.9% (9). The study recommended intensification of media campaigns and advocacy efforts for COVID-19 vaccination, targeting the southeastern and northwestern regions and specific demographic groups. Individuals with no formal education and those in the 18–29 age bracket were identified as less likely to have been vaccinated % (9). Various communication channels, including government sources, mass media, and healthcare workers, were recommended to positively influence citizens' decisions regarding COVID-19 vaccines acceptance across the diverse demographic and geographic contexts.

### 3.4 Top of form

A study on COVID-19 vaccine landscape in Nigeria revealed that majority of participants expressed receptiveness to the COVID-19 vaccination (28). Specifically, the western and northern regions exhibited the highest levels of vaccine acceptance and hesitancy, respectively. The research indicated that 2020 marked the year with the highest level of vaccination acceptance compared to subsequent years. It identified key factors associated with vaccine uptake, emphasizing a significant connection between being male and risk perception which should be taken into consideration by policymakers when formulating vaccination policies. On regional disparities, the study suggested focused approaches for enhancing vaccine acceptance including increased sensitization efforts targeting local authorities and the dissemination of detailed information about the COVID-19 vaccine, particularly in the northern, southeastern, and north-central states.

Another study applied a behavioral lens to study the factors influencing COVID-19 vaccination uptake among healthcare workers (HCWs) in Nigeria using data gathered from an online survey conducted in July 2021 among Nigerian HCWs aged 18 and older (24). Multivariate logistic regression analyses were employed to scrutinize the predictors of receiving two doses of a COVID-19 vaccine. One-third of HCWs reported receiving two doses of a COVID-19 vaccine. Thirty-two per cent (32%) of them reported very easy access to a COVID-19 vaccination. In contrast, motivation levels were relatively high: 69% of HCWs reported that a COVID-19 vaccine was very important for their health. The study highlighted the necessity of simplifying the process for HCWs to access COVID-19 vaccinations. Given the central role that HCWs play in managing and mitigating the impact of the COVID-19 pandemic in Nigeria, removing barriers to vaccine access was identified as crucial.

Another study provided insights into the acceptance rate of COVID-19 vaccines in Nigeria as well as the factors influencing non-acceptance (29). Their examination of existing literature discovered a diverse range of acceptance rates among adults in Nigeria, ranging from 20.0% to 58.2% across the six geopolitical zones (30). They identified the impact of propaganda, concerns related to adverse effects, and the influence of conspiracy theories as reasons for the reluctance to accept COVID-19 vaccines. The researchers advocated for targeted efforts aimed at addressing the specific expressed concerns and issues that contribute to vaccine hesitancy among the population. These efforts may involve public health campaigns, educational initiatives, and community engagement to counter misinformation, allay fears regarding adverse effects, and debunk conspiracy theories associated with COVID-19 vaccines. The study emphasized the criticality of understanding and addressing the nuanced factors contributing to vaccine hesitancy in Nigeria including multifaceted interventions that address the unique challenges faced by different regions and demographics. This can inform a comprehensive strategy to improve the overall acceptance rate of COVID-19 vaccines in the country.

### 3.5 Enablers, barriers, and facilitators of COVID 19 vaccine uptake in Nigeria

On the side of the community and individual levels, various factors that promoted the intention for vaccine uptake were reported in thirteen studies. Across studies these were grouped as confidence in the COVID-19 vaccine and the desire to protect others, e.g., family and community members. Only few respondents identified acceptance of the vaccine as a public responsibility (17, 29). In addition, other reasons related to being better informed about COVID-19 such as awareness of the possible side effects of the vaccine; increased COVID-19 vaccine education, observing others receive the COVID-19 vaccine; having free access to the COVID-19 vaccine; receiving a vaccine certificate; prior diagnosis of COVID-19; having a positive perception of the COVID-19 vaccine; having access to media; having a high perceived susceptibility of contracting COVID; the presence of comorbidities; COVID-19 vaccines being recommended by HCWs and it being for self-protection (17, 29).

The enabling factors from the policy-makers and the health systems were largely on vaccine availability and policies. The following were identified by the studies: the support from partners in ensuring availability of the vaccine, the stewardship of the NPSCMP and the coordinating MDA – the Nigeria Centre for Disease Prevention and Control (NCDC), the surveillance systems, the availability of health facilities, human resources, health education and monitoring and evaluation systems (17).

A range of factors contribute to low uptake and equitable access to COVID-19 vaccination in Nigeria (see Table 2): religious influences, cultural Inclination, myths, residential segregation, cultural inclination, myths, distance to health facilities, finances—transportation challenges, lack of faith in vaccines, fear of side effects and complications, incorrect information/education, poor level of awareness, never being offered before, low literacy levels on the community and individual level, while the national and health systems level, it was found that poor infrastructure, change in waiting time, funding challenges, vaccine shortage and complexities in Nigerian distribution channels were the barriers to COVID-19 vaccination uptake and equitable access (17, 29). As summarized in Table 5, these enablers were identified at different levels of the vaccination landscape.

Studies also showed that religious beliefs (e.g., the vaccine contains the mark of the beast), and would not accept the COVID-19 vaccine from Western or European countries, which resulted in poor vaccine uptake (17, 29). One study reported on participants being afraid of needles, having a negative perception of the vaccine (17). Tight work schedules, vaccine effectiveness, and being pregnant (29). Vaccine-related misconceptions include the idea that being injected with COVID-19 vaccines will affect one's reproductive system, such as causing barrenness in women and impotence in men. Furthermore, vaccines are made to make people foolish and are intended to kill the African population (17, 29). The structural barriers identified were long queues at vaccination centers, accompanied by vaccine shortages and proximity to a vaccination center.

### 3.6 Factors associated with COVID-19 vaccine hesitancy in Nigeria

Fifteen papers were included in the final analysis based on the inclusion and exclusion criteria for this research on COVID-19 hesitance in Nigeria. There were differences in terms of the study region, study design, and study participants. In terms of the study region, the studies were conducted across different regions and states in Nigeria (18, 19, 21, 24, 25, 17, 26, 27). Majority of the studies were conducted in the south southern region, followed by south west and then northcentral and northeast. In terms of study design and participants, 60% of the studies were cross-sectional quantitative and qualitative (21, 25, 17, 31). The hospital-based studies were conducted on health workers (7%) and patients (13%), respectively while 20% of the studies were school-based cross sectional-studies conducted among university staff and students (6, 9, 18, 19, 21, 24, 25, 17, 26, 28–32). Overall, the average hesitancy rate found in review ranged from 51% to 68.5%.

COVID-19 vaccine hesitancy in Nigeria is influenced by various factors that contribute to individuals' reluctance or refusal to receive COVID-19 vaccinations. These factors as identified in the studies were multifaceted and varied across different demographic groups, regions, and communities across the country. Findings from the review revealed the following socio-demographic factors: gender, participant's age, educational level, religious and cultural views, rural residence and urban slum as well as the participant's income level (9, 24, 30–32). The review also identified vaccine-specific determinants which included lack of belief in the existence of COVID-19 (24), lack of trust in the safety and effectiveness of the vaccines as well as lack of trust in the Nigerian public health systems (9).

In Nigeria, a significant factor driving hesitancy against COVID-19 vaccination is rooted in a pervasive disbelief regarding the very existence and severity of the disease. This scepticism manifests in a tendency to downplay the gravity of COVID-19, leading to an underestimation of the importance of getting vaccinated (32). The scepticism surrounding COVID-19 has created an environment where individuals, influenced by misinformation or lack of awareness, question the necessity of vaccination. Skeptics possess the perception that the virus is less severe than it is in reality. The study further revealed that the reluctance to accept the severity of the disease has a direct impact on the willingness to receive the COVID-19 vaccine. If individuals do not perceive the disease as a significant threat, they may view vaccination as unnecessary or even unwarranted. Obviously, this perception creates a hurdle for public health efforts, as widespread vaccine coverage is critical for controlling the spread of the virus. The scepticism-induced hesitancy presents a nuanced challenge for public health campaigns, as it requires addressing not only concerns about the vaccine itself, but also the underlying doubts about the severity of COVID-19 (24).

Further, the widespread vaccine hesitancy in Nigeria is significantly driven by perceived concerns surrounding the efficacy and safety of the COVID-19 vaccine (9, 24). A substantial number of respondents expressed apprehension regarding the potential side effects of the vaccine, contributing to a hesitancy to get vaccinated (29). One key aspect of these concerns revolves around the fear that the COVID-19 vaccine could induce adverse effects on individual health including reproductive functions (24).

The spread of conspiracy theories through both mainstream and social media has notable effects on COVID-19 vaccine hesitancy in Nigeria, influencing public perception and decision-making in several ways. Conspiracy theories often promote distrust in the effectiveness and safety of vaccines, leading individuals to question the credibility of health authorities. The dissemination of such theories through various media channels has contributed to a decline in trust in both the COVID-19 vaccines and the institutions promoting them. This erosion of trust significantly impact vaccine hesitancy, as people may be hesitant to receive a vaccine they perceive as potentially unsafe or untrustworthy (32). Conspiracy theories generate and amplify misinformation about the COVID-19 vaccines; false claims and myths, such as that the vaccines cause severe side effects or contain harmful substances, spread easily through mainstream and social media platforms. This misinformation creates apprehension and fear, deterring individuals from getting vaccinated. In Nigeria, where cultural or religious beliefs influence vaccine hesitancy, conspiracy theories can further amplify these barriers. The combination of traditional suspicions and the influx of misinformation from media sources can reinforce hesitancy among certain communities (33).

The Nigerian public health system is under intense strains due to accumulated years of poor budgetary allocations, poor infrastructure and equipment, dysfunctional systems, depleting health workforce due to emigration of trained personnel and mismanagement (24). Additionally, the supply chain logistics management system is poorly organized leading to inefficiencies, wastes and overall, under performance manifested in unpredictable supply chains compounded by the national lack of capacity for local vaccine production.

The reluctance of Nigerians to accept or take the COVID-19 vaccine is strongly linked with a pervasive mistrust in the government's responses to the pandemic, including suspicions about the possible influence of foreign political economic interests (31). A key aspect of this scepticism revolves around the perception of COVID-19 as a hoax orchestrated by foreign nations, particularly emphasizing a belief that the Nigerian government is exploiting the situation for personal pecuniary gains. This perception not only questions the legitimacy of the disease but also raises concerns about the motives behind the vaccination efforts, presenting a multifaceted challenge for public health interventions (24, 30–32).

The perception among some Nigerians that COVID-19 vaccination efforts were unnecessary stemmed from a belief in the efficacy of alternative preventive medicines, particularly chloroquine (24). Many individuals, especially older adults, held the view that chloroquine, traditionally used to treat endemic malaria, could provide collateral immunity against COVID-19. This belief was rooted in the idea that the long-term use of drugs such as chloroquine for malaria control might be a contributing factor to the comparatively low reported cases of COVID-19 infection in Nigeria (9). Additionally, the lack of direct personal experience or knowledge of someone with a severe COVID-19 infection further reinforced this sentiment (9, 24).

## 4 Discussion

Our review aimed to assess the prevalence of vaccine acceptance and uptake in Nigeria and proffer actionable recommendations to curb hesitancy and enhance timely and equitable coverage of COVID-19 vaccine in Nigeria. We found that the studies showed an average range of 9% to 24% rate of uptake of COVID-19 vaccine. On the other hand, the magnitude of vaccine hesitance in Nigeria was found to range from 51% to 68.5% (33). Low uptake and high hesitancy of COVID-19 vaccine was found across studies in different regions. The low uptake and high hesitancy found in the review may be associated with differences in region, access to information and locations which correlates with the studies conducted in the United States which revealed ethnic, access to vaccine information and location disparities in uptake of COVID 19 (12, 13). This result is also consistent with the finding from a study in the Northern and Eastern parts of Nigeria which reported a sub-optimal coverage and high hesitancy rate (10, 11).

Further, the study also found supply side factors that could impede equitable access and uptake of the vaccines such as shortage of vaccines, complexities of the Nigerian vaccine distribution channels, funding, inadequate infrastructures, shortage of human resources for health; this was corroborated by another Nigerian study (14). On the other hand, the review found other factors at the individual and community levels that hinder vaccine uptake while promoting hesitancy. These factors were being within the age group 18–30 and 40 years and above; they are less likely to take up the vaccination, especially females. We also found that rural dwellers are less likely to take the vaccine and more hesitant than those in the urban centers. Similarly, low literacy level and poor access to information were found to be associated with hesitancy and low uptake of the vaccine. This finding is consistent with a study by Pathway 2023 (33). Educational level is a factor associated with vaccine uptake and hesitancy. This is an important factor that should be considered in the development of contextual strategies for vaccine by the Nigerian health systems. This result corroborates with the result of a study conducted in low and middle income countries including Africa, which reported that education level was a factor associated with vaccine acceptance/uptake and marginally with vaccine hesitancy, stating that people with above secondary school are more likely to access COVID 19 vaccination (34).

The review also identified vaccine-specific and health systems determinants which contribute to high COVID-19 vaccine hesitancy and low uptake. These factors are (1) lack of belief in the existence of the virus, (2) lack of trust in the safety and efficacy of the vaccines, (3) lack of trust in the Nigerian health systems, (4) misinformation and myths, as well as the perception among that COVID-19 vaccination efforts were unnecessary fuelled by a belief in the (5) efficacy of alternative preventive medicines, particularly chloroquine. Many individuals, especially older adults, held the view that chloroquine, traditionally used to treat endemic malaria, could provide collateral immunity against COVID-19. This also correlates with finding of a study different African countries which reported the speculation that, in some places, very high doses of chloroquine and its derivative are used for chemoprevention and treatment of COVID-19 (35). Such practices must be discouraged through intense and sustained education as continuation may lead possibly to serious health implications.

According to studies in this review, uncertainties around vaccination safety and efficacy in the population can be minimized by increasing the level of trust in vaccines and particularly in institutions (6). Also, people with varied levels of institutional trust followed different levels of vaccine acceptance over time. Trust in government especially the health system, is a necessary component of vaccine acceptance and a key factor in vaccine hesitancy since all other efforts, including large-scale vaccination campaigns to highlight the high efficacy of vaccines in controlling epidemics seem to depend on it.

It was revealed that COVID-19 vaccines were regarded as “mark of the devil” and as weapon of destruction of Africans by the western countries (29). Other factors include perceived less severity of COVID-19 and less vulnerability to the disease. The study recommends that interventions geared towards clearing the misconceptions about COVID-19 vaccination should involve religious leaders like pastors, reverend fathers, and imams to help educate their followers about the importance of COVID-19 vaccination and the falsehood about conspiracy theories regarding COVID-19. Traditional rulers and other influential people in the communities should also be involved for the purpose of persuading their subjects to take COVID-19 vaccination.

### 4.1 Strategies for equitable distribution and delivery of COVID-19 vaccines

More intensified efforts through every appropriate media should be made towards sharing accurate information regarding the COVID-19 vaccines, their importance, and the negative implications of refusing vaccination.

Efforts to combat vaccine hesitancy should involve targeted communication strategies that emphasize the real and potentially severe consequences of the disease while countering misinformation. Additionally, public health initiatives should focus on increasing awareness and understanding of the virus, its impact on individuals and communities, and the role of vaccination in preventing its spread. The prevailing skepticism regarding the existence and severity of COVID-19 in Nigeria should be addressed through the provision of accurate information to dispel myths, and underscore the importance of vaccination in safeguarding individual and community health.

Emphasizing the importance of COVID-19 vaccination as a specific and effective preventive measure, supported by rigorous scientific research and global health recommendations, can help dispel misconceptions and build confidence in the safety and efficacy of the vaccines.

Public health campaigns, educational initiatives, and nuanced community engagement efforts should address the issue of misinformation by increasing awareness about the impact of COVID-19. By fostering a better understanding of the disease and the benefits of vaccination, these measures would reduce skepticism, ultimately contributing to a higher acceptance of COVID-19 vaccines in Nigeria.

Public health campaigns should be designed to address specific concerns raised by conspiracy theories and ensure that accurate information reaches diverse audiences through both mainstream and social media platforms. Addressing the concerns requires targeted communication strategies, community engagement, and efforts to rebuild trust in both the vaccine and the broader systems involved in pandemic management.

### 4.2 Strengths and limitations

The strengths of the study include but not limited to being one of such studies to bring together studies and key factors of COVID-19 vaccine hesitancy in Nigeria. It identifies gaps in current research, and informs policy and practice in preparing the response for future epidemics and pandemics. A good number of the studies reviewed was qualitative which is capable of extending knowledge and understanding complex and context-specific phenomena, such as vaccine hesitancy.

However, most studies included in this review were cross-sectional. Since cross-sectional studies collect data at a given time, they may provide different results if another time frame was chosen. Additionally, potential selection bias due to inclusion and exclusion criteria limited the number of articles included in the review.

## 5 Conclusion

There was very low COVID-19 vaccination acceptance and uptake among Nigerians. Age, living in a rural area, low educational attainment and gender were some of the demographic factors associated with COVID-19 vaccine uptake and hesitancy across the country.

Lack of adequate information, misinformation, conspiracy theories, poor access, vaccine scarcity, lack of trust on efficacy and fear of possible side effects were some identified vaccine-specific deterrents to uptake of the vaccine.

There is obvious need for policy makers, health care providers and relevant stakeholders to appreciate the contextual drivers of the prevalent hesitancy and low uptake of COVID-19 vaccines in Nigeria in order to develop and implement evidence-based strategies and policies that will target different population groups and demographics to improve uptake and reduce hesitancy among the citizens.

## Data Availability

The original contributions presented in the study are included in the article/Supplementary Material, further inquiries can be directed to the corresponding author.
